# Use of the relative release index for histamine in LAD2 cells to evaluate the potential anaphylactoid effects of drugs

**DOI:** 10.1038/s41598-017-14224-z

**Published:** 2017-10-20

**Authors:** Shengli Han, Yanni Lv, Liyun Kong, Delu Che, Rui Liu, Jia Fu, Jiao Cao, Jue Wang, Cheng Wang, Huaizhen He, Tao Zhang, Xinzhong Dong, Langchong He

**Affiliations:** 1 School of Pharmacy, Xi’an Jiaotong University, Xi’an, 710061 China; 2 The Solomon H. Snyder Department of Neuroscience, Johns Hopkins University, School of Medicine, Baltimore, Maryland 21205 USA; 3 Department of Pharmaceutical Science, School of Pharmacy, University of Wisconsin-Madison, Madison, WI 53705 USA

## Abstract

Anaphylactoid reactions are common clinical acute adverse drug reactions that can exacerbate a patient’s condition and produce effects that may become life-threatening. Therefore, it is important to establish a novel method to evaluate drugs for anaphylactoid reactions. In this study, we developed a sensitive and rapid method to detect histamine release from LAD2 cells using liquid chromatography-tandem mass spectrometry (LC-MS/MS) and constructed a relative release index based on various release curve parameters, including allergen release time and sudden change rate, to evaluate the potential and strength of allergen-induced anaphylactoid reactions. This LAD2 release model was used to evaluate anaphylactoid reactions induced by ciprofloxacin, norfloxacin, lomefloxacin, moxifloxacin, and baicalin. The results positively correlated with those obtained with an Evans blue ear test and negatively correlated with the Ca^2+^ influx EC_50_. In summary, the current study established a novel *in vitro* method to analyze the properties of histamine release from LAD2 cells and characterize the sensitization and strength of sensitization of drugs or components that may induce anaphylactoid reactions.

## Introduction

Anaphylactoid reactions are common clinical acute adverse drug reactions that can exacerbate a patient’s condition and produce effects that may become life-threatening^1–3^. According to early studies, unlike in the usual type I hypersensitivity reaction, in anaphylactoid reactions, the main mechanism involves the direct stimulation of IgE-R on mast cells or basophils or the activation of complement by allergens via an alternative pathway^4,5^. These reactions lead to the release of anaphylactic mediators such as histamine and β-hexosaminidase^6^. A recent study by Dong *et al*. reported that *Mrgpr*X2, a specific membrane receptor on human mast cells, induces anaphylactoid reactions. Some medications, such as ciprofloxacin and rocuronium, induce *Mrgpr*X2-mediated degranulation, thereby causing anaphylactoid reactions^7^.

Specific receptors that are overexpressed on mast cells, such as IgE-R and *Mrgpr*X2, are direct targets for triggering anaphylactoid reactions. LAD2, Ku812, and HMC-1 are three human mast cell lines commonly employed to study anaphylactic and anaphylactoid reactions^8–11^. In particular, LAD2 is often used because its biological properties are identical to those of primary human mast cells, including an abundance of IgE-R and overexpression of the *Mrgpr*X2 receptor^12^. Despite the availability of these cell lines, allergen screening and evaluation remains a serious clinical problem. In general, type I hypersensitivity reactions are assessed using active systemic allergy tests and passive cutaneous anaphylaxis models^13,14^. However, the mouse ear (Evans blue) and local toe swelling tests are the most common evaluation methods because enhanced vascular permeability is a major pathological characteristic of anaphylactoid reactions^15,16^. The release of anaphylactic mediators, including histamine and β-hexosaminidase, is commonly assessed by ELISA^17,18^, although there are difficulties associated with the detection of histamine and β-hexosaminidase, including but not limited to low sensitivity, poor reproducibility, and a complicated protocol.

**Figure 1:**
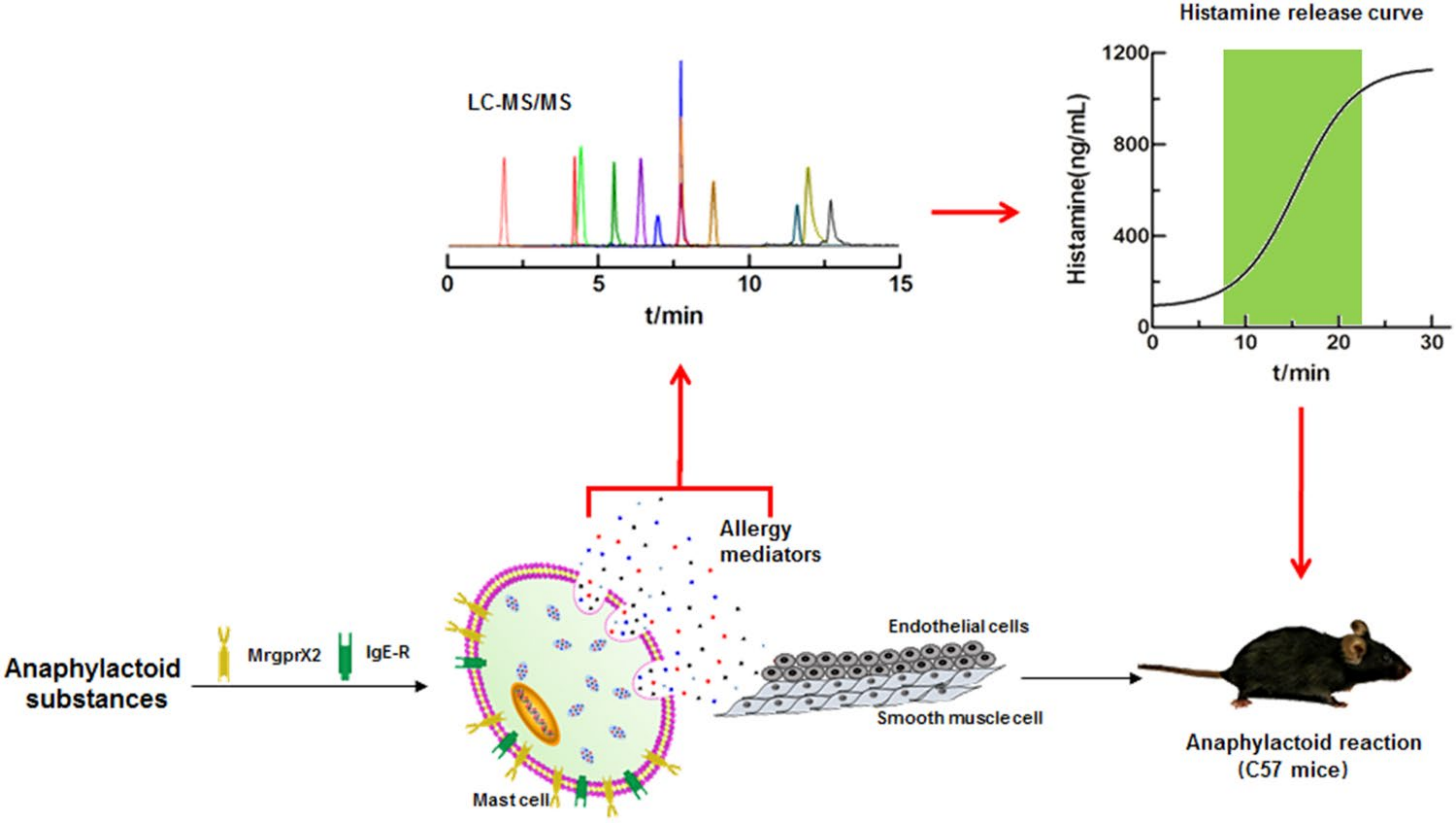
Schematic of the histamine release curve used to evaluate anaphylactoid reactions.  Allergy mediators detected by LC-MS/MS to obtain the histamine release curve to evaluate anaphylactoid reactions;  Anaphylactoid substances that act on *Mrgpr*X2 or IgE-R on mast cells, induce the release of allergy mediators, and eventually cause anaphylactoid reactions via endothelial cells or smooth muscle cells.

In this study, we established a sensitive and rapid method to detect histamine release from LAD2 cells using liquid chromatography-tandem mass spectrometry (LC-MS/MS) and constructed a relative release index (RRI) based on various release curve parameters, including allergen release time and sudden change rate, to evaluate the potential and strength of allergen-induced anaphylactoid reactions. Our strategy for the evaluation of anaphylactoid reactions via this LAD2 cell release model is presented in Fig. 1.

## Results and Discussion

**Figure 2:**
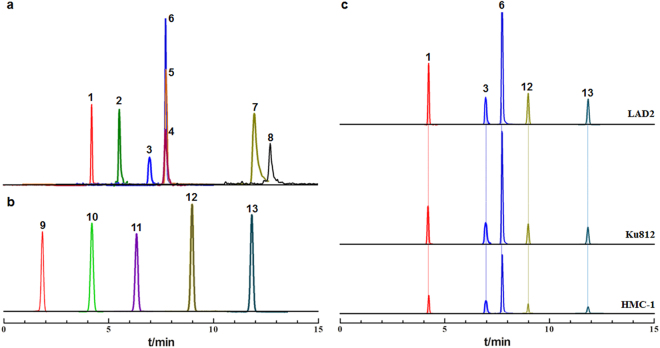
Typical LC-MS/MS chromatograms of allergy mediators. (**a**) Eight allergy mediators with low molecular weights (1: serotonin (5-HT), 2: thromboxane B2 (TXB2), 3: prostaglandin E2 (PGE2), 4: prostaglandin D2 (PGD2), 5: methylhistamine (MHA), 6: histamine (HA), 7: leukotriene E4 (LTE4), 8: platelet activating factor (PAF)); (**b**) Five allergy mediators with high molecular weights (9: interleukin 4 (IL-4), 10: interleukin 6 (IL-6), 11: interleukin 8 (IL-8), 12: β-hexosaminidase, 13: tumor necrosis factor-α (TNF-α)); (**c**). Allergy mediators released by three types of human mast cells (LAD2, Ku812, and HMC-1 cells).

We established an LC-MS/MS method to simultaneously detect 13 anaphylactic mediators, specifically eight small molecules and five macromolecules (Fig. 2a and b). In addition, a system suitability test that examined the linearity, specificity, detection limit, reproducibility, and precision of the methods used to analyze the 13 anaphylactic mediators indicated that the results met the quantitative analysis requirements. The system suitability results are detailed in Supplementary Table 1. Using the aforementioned method to detect anaphylactic mediator release from three human mast cell lines (LAD2, Ku812 and HMC-1), five anaphylactic mediators were identified within the effective release time (<30 min) (Fig. 2c). Among these mediators, histamine, serotonin, and β-hexosaminidase release was significantly higher than that of PGE2 and TNF-α. Moreover, the five anaphylactic mediators were released in significantly greater amounts by LAD2 cells than by Ku812 and HMC-1 cells, which recapitulated the more sensitive properties of anaphylactic mediator release from LAD2 cells following allergen stimulation. The amounts of anaphylactic mediators released are shown in Supplementary Table 1.

**Figure 3:**
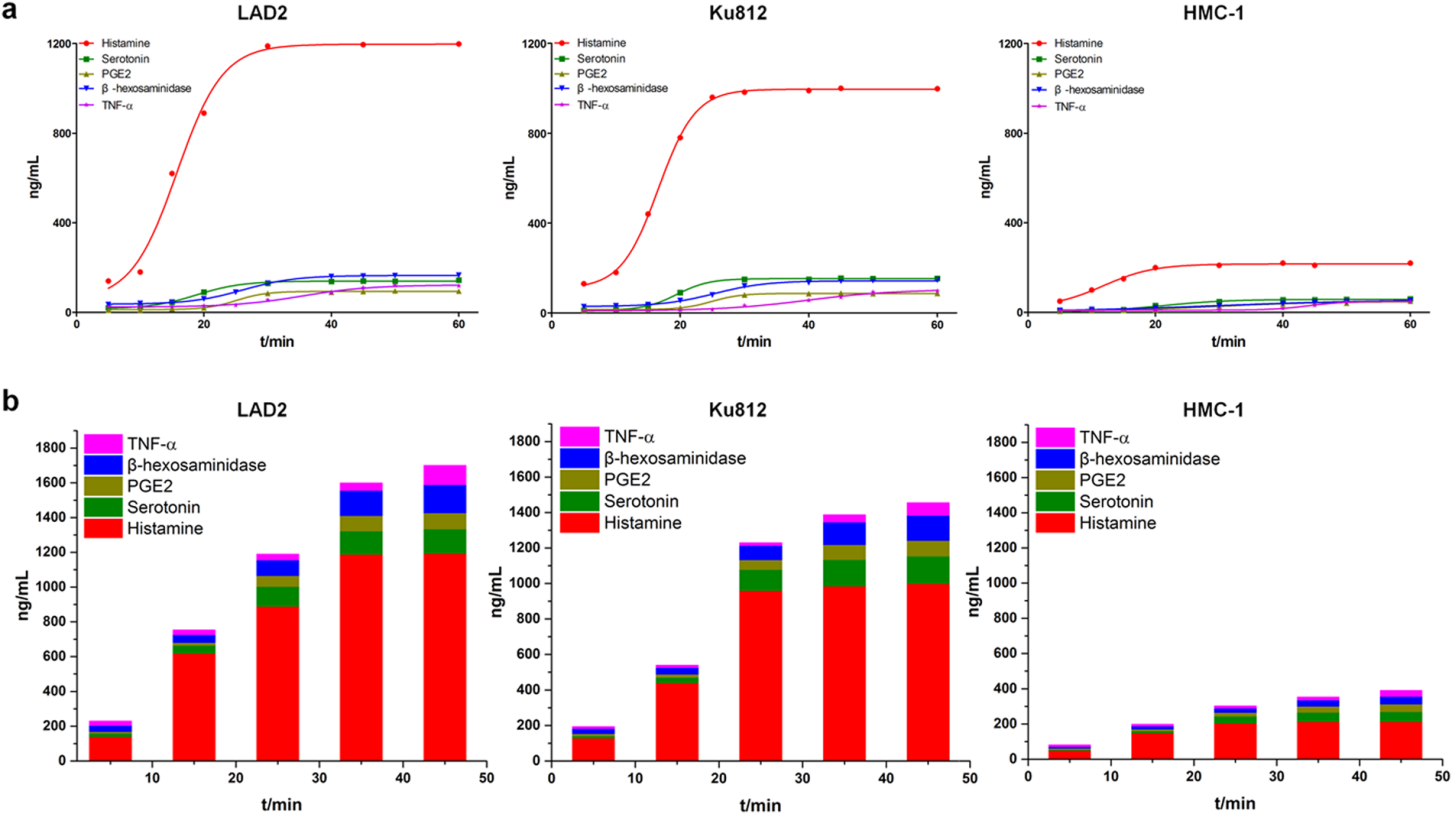
Characteristics of allergy mediators released from LAD2, Ku812, and HMC-1 cells. (**a**) Release curves (Cx-t) for five allergy mediators, including the release time (t). Cx: the release concentrations of histamine (HA), serotonin (5-HT), prostaglandin E2 (PGE2), β-hexosaminidase and tumor necrosis factor-α (TNF-α)); (**b**) Total release concentrations (Ct) of five allergy mediators, including the release time (t).

The time-effect release curves of the five anaphylactic mediators were determined by stimulating the three cell lines with the model drug compound 48/80. Histamine was released at a significantly higher concentration than the other four mediators between 0–60 min (Fig. 3a and Supplementary Table 2). Of the three cell types, LAD2 cells released the highest concentration of histamine (Fig. 3a). In addition, mediator release was measured at five different time points for all three cell lines, and the total mediator concentration from LAD2 cells was greater than that from Ku812 cells and markedly greater than HMC-1 cells (Fig. 3b), demonstrating a positive correlation between the subtypes of anaphylactoid reaction-inducing receptors on the three cell lines and receptor numbers (Supplementary Table 3). These findings also indicated that LAD2 cells were not only enriched in anaphylactoid reaction-related receptor subtypes but also released large amounts of histamine. Recently, the LAD2 cell line has been widely used in anaphylaxis and anaphylactoid reaction studies and is a promising tool for measuring the ligand/receptor-induced (e.g., *Mrgpr*X2, IgE-R) mast cell release of inflammatory mediators. Therefore, LAD2 was used as a model cell line to ascertain the potential and the extent of drug-induced anaphylactoid reactions via the construction of a histamine release curve.

**Figure 4:**
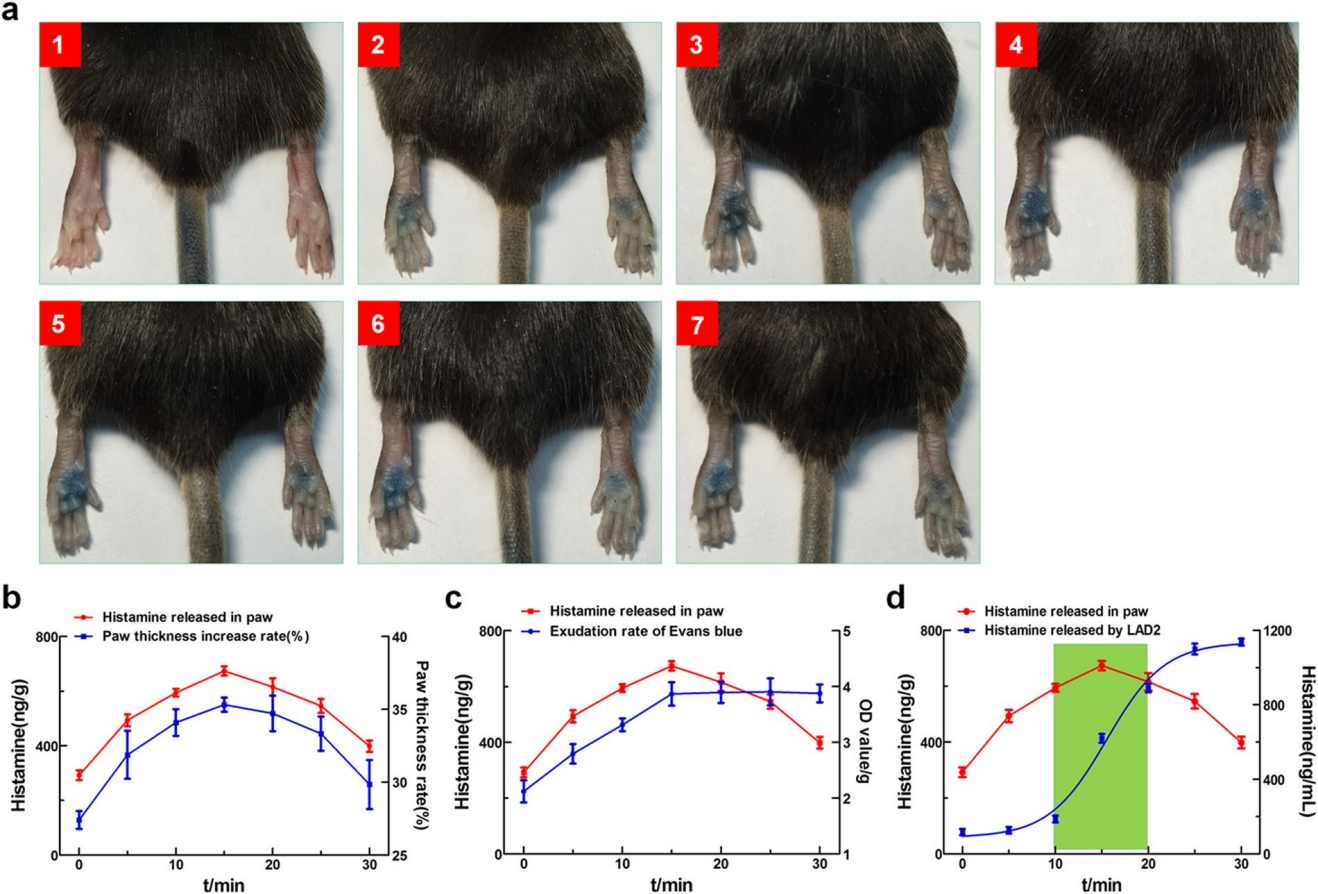
LAD2 cell histamine release correlates with anaphylactoid reactions in C57 mice. (**a**) Paw thickness and Evans blue exudation in C57 mice after compound 48/80 injection at different time points (0, 5, 10, 15, 20, 25 and 30 min); (**b**) The correlation between the paw thickness rate and local histamine released in C57 mice after compound 48/80 injection at different time points (0, 5, 10, 15, 20, 25 and 30 min); (**c**) The correlation between the Evans blue exudation rate and local histamine released in C57 mice after compound 48/80 injection at different time points (0, 5, 10, 15, 20, 25 and 30 min); (**d**) The correlation between local histamine released in C57 mice and LAD2 cell histamine release.

Degranulation is a basic function of mast cells following activation by certain drugs, particularly fluoroquinolones. Histamine is the key mediator in mast cell granules. Histamine release induces vascular permeability and inflammatory infiltrates following local or systemic pseudo-allergic reactions. A local tissue swelling assay (paw swelling) in mice at 0, 5, 10, 15, 20, 25, and 30 min post-stimulation with compound 48/80 showed that maximum swelling occurred at 15 min post-stimulation and then reduced gradually (Fig. 4a and b). The detection of local histamine release in the paw at each time point indicated that the degree of paw swelling and the Evans blue exudation rate were both associated with the amount of local histamine release (Fig. 4b and c). Therefore, there are close relationships between tissue histamine release and anaphylactoid reaction. Furthermore, we compared histamine release from tissue and from LAD2 cells. The histamine release curve for LAD2 cells indicated a release time of 14.6 min, with a sharp increase between 9 and 20.5 min (Fig. 4d). Similarly, the peak of local tissue histamine release was 15 min, with an increase similar to that observed for LAD2 cells (Fig. 4d). Based on these results, there was a positive correlation between the maximum interval of tissue histamine release and histamine release in exponentially growing LAD2 cells. Thus, the *in vitro* properties of histamine reflect *in vivo* anaphylactoid reactions.

**Figure 5:**
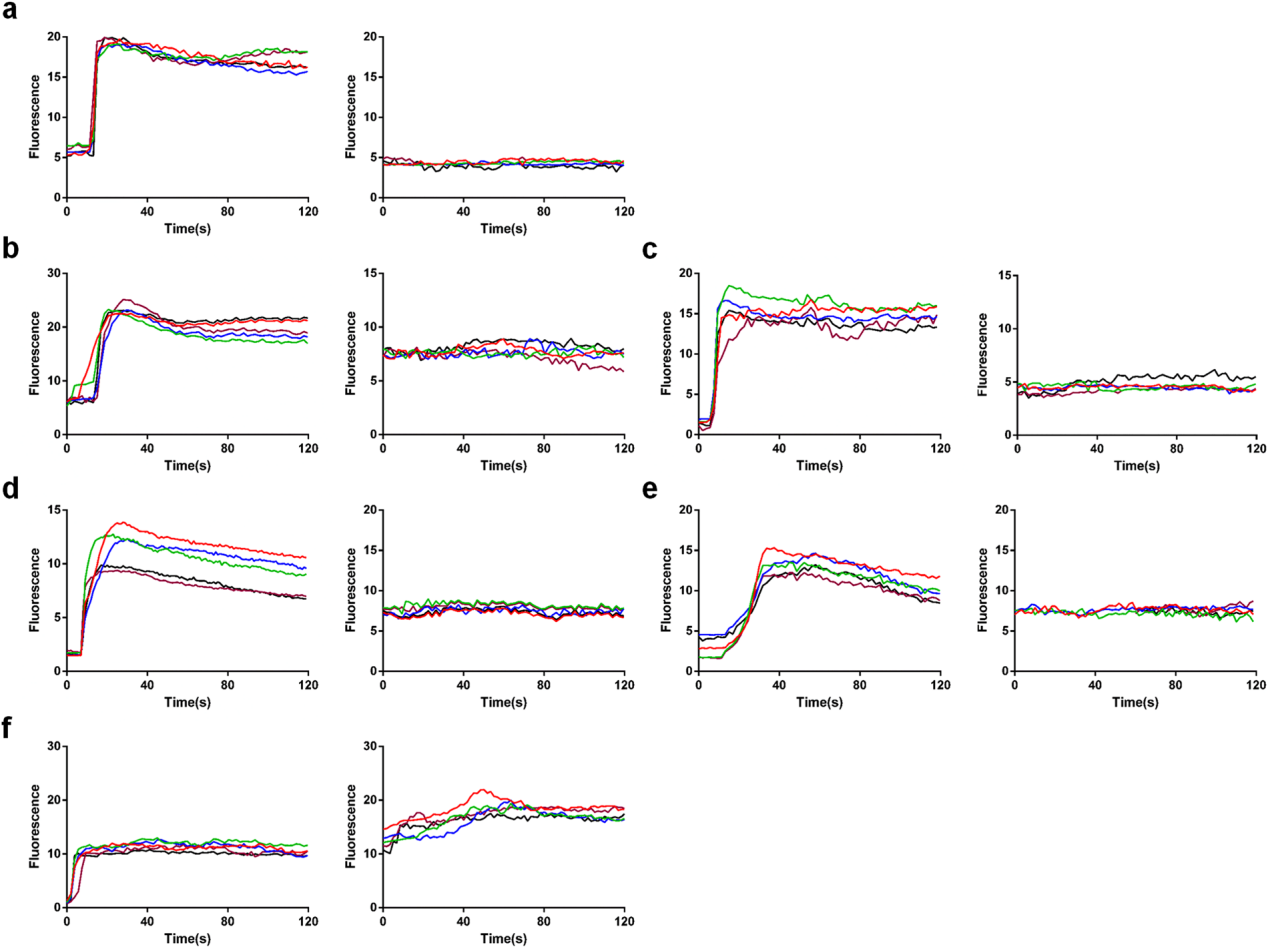
The mechanism of anaphylactoid substances acting on LAD2 cells. The Ca^2+^ influx of compound 48/80 (**a**), ciprofloxacin (**b**), moxifloxacin (**c**), norfloxacin (**d**), lomefloxacin (**e**), and baicalin (**f**) in LAD2 cells and *Mrgpr*X2-knockdown LAD2 cells.

The results of the expression of MrgprX2 and the knockdown efficiency of MrgprX2 were investigated (Supplementary Figure 1). The knockdown efficiency was good. Therefore, in the present study 11 substances were screened at a concentration of 100 μg/mL by performing calcium imaging using *Mrgpr*X2-HEK293 cells and HEK293 cells (Supplementary Figure 2). Six substances (compound 48/80, ciprofloxacin, moxifloxacin, lomefloxacin, norfloxacin and baicalin) representing 3 different classes of molecules that induce intracellular calcium influx via *Mrgpr*X2 were selected for further evaluation (Fig. 5a–f). Next, calcium imaging was performed for these six substances in LAD2 cells and *Mrgpr*X2 knockdown LAD2 cells. Silencing of *Mrgpr*X2 expression in LAD2 cells caused intracellular calcium influx to disappear. We concluded that the induction of mast cell activation by compound 48/80, ciprofloxacin, moxifloxacin, lomefloxacin, norfloxacin and baicalin was mediated by *Mrgpr*X2. Thus, we applied the *in vitro* LC-MS/MS method to analyze the *Mrgpr*X2-mediated anaphylactoid reactions triggered by these substances.

**Figure 6:**
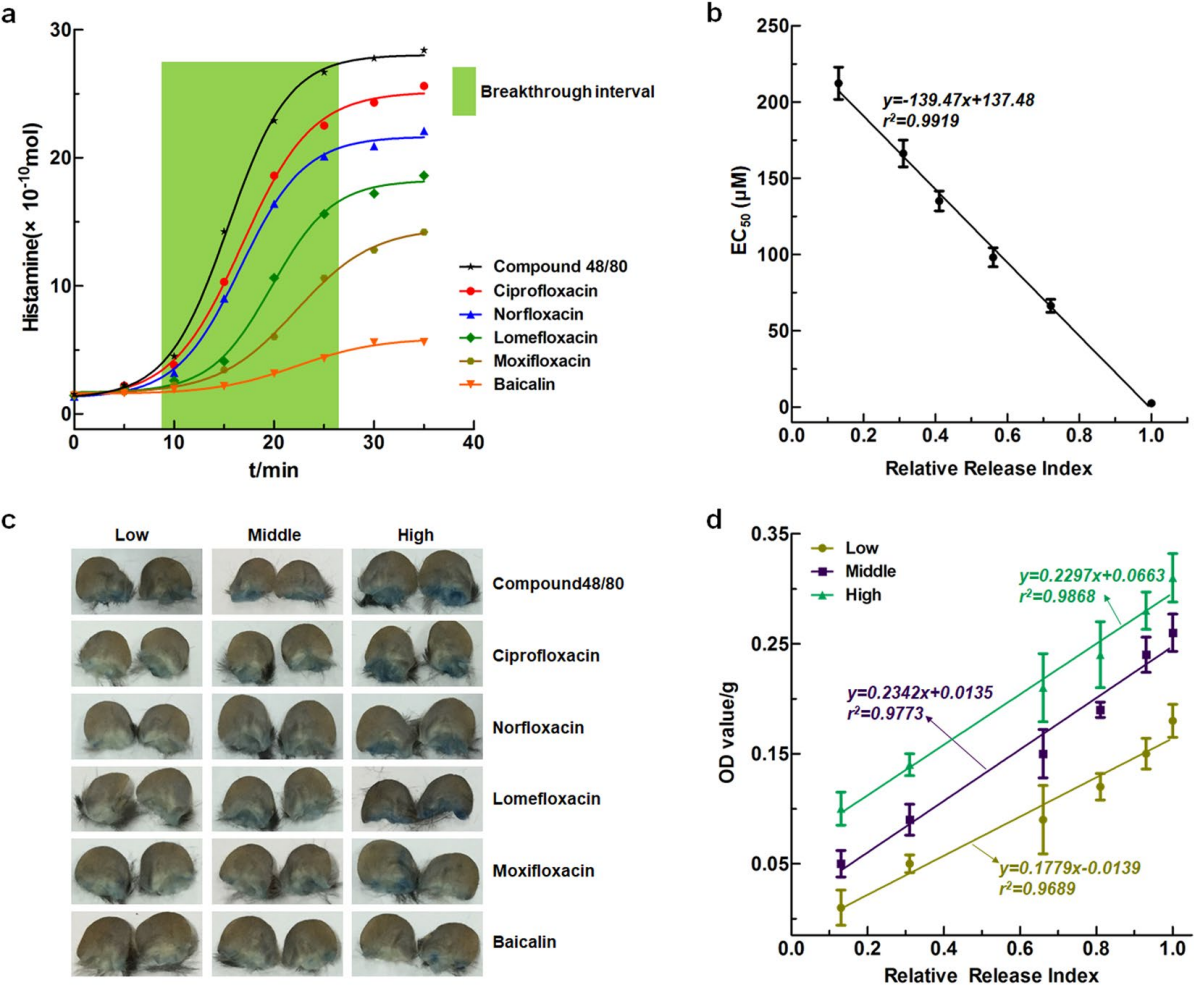
The RRI of histamine for LAD2 cells correlates with the anaphylactoid effects of drugs in C57 mice. (**a**) The histamine release curves (Cx-t) of six potential anaphylactoid drugs in LAD2 cells, including the release time (t). Cx: the released concentrations of compound 48/80 (as a positive control), ciprofloxacin, norfloxacin, lomefloxacin, moxifloxacin and baicalin; (**b**) The correlation between the histamine RRI and the EC_50_ values (concentrations giving half-maximal effect) of six drugs (compound 48/80, ciprofloxacin, norfloxacin, lomefloxacin, moxifloxacin and baicalin); (**c**) Evans blue ear extravasation results following stimulation by six potential allergic components; (**d**) The correlation between the histamine RRI and the OD values from the Evans blue ear extravasation experiment.

Anaphylactoid reactions induced by ciprofloxacin, norfloxacin, lomefloxacin, moxifloxacin, and baicalin are mediated through *Mrgpr*X2. Therefore, this study sought to use the histamine release curve for LAD2 cells (release time, release breakthrough velocity, release index (RI) and RRI to characterize drug-induced anaphylactoid reactions. First, LAD2 cells were stimulated with the five drugs and compound 48/80, and histamine release was assessed at different time points to obtain time-effect curves, designated histamine release curves (Fig. 6a), which were used to determine the corresponding histamine release time, release breakthrough velocity, RI and RRI of each drug. To clarify the function of the RRI value we obtained, we subsequently investigated the relationships between RRI values and anaphylactoid effects both *in vitro* and *in vivo*. Correlation analysis showed a strong negative relationship (r^2^ = 0.9919) between RRI and the EC_50_ of Ca^2+^ influx in LAD2 cells induced by these substances (Fig. 6b). The higher the EC_50_ value of a compound, the better effect it induces, and in this study, a higher EC_50_ value indicated the easier induction of an anaphylactoid reaction. The drug with the highest RRI tended to release the highest amount of histamine and thus induced an anaphylactoid reaction with a lower dose. Additionally, the anaphylactoid reaction-inducing effects of compound 48/80 and the five antibiotics were examined *in vivo* using the Evans blue ear test. Low, intermediate, and high concentrations of all six drugs significantly induced anaphylactoid reactions in mice in a dose-dependent manner (Fig. 6c). Furthermore, the RRI value of each drug was calculated according to equations (1–3), outlined in the Methods section. To clarify the function of the RRI value we obtained, we subsequently investigated the relationships between RRI values and anaphylactoid effects. Evans blue exudation induced by the tested substances was positively correlated with the RRI (r^2^ = 0.9689, 0.9773, and 0.9868, respectively) (Fig. 6d and Supplementary Table 4). Although rodents are relatively insensitive to histamine^19^, histamine was the best mediator for characterizing degranulation *in vitro*. Therefore, we used the RRI of the LAD2 cell release model established *in vitro* to evaluate anaphylactoid reactions *in vivo*.

## Conclusion

In this study, an LC-MS/MS method was established and used to analyze the anaphylactoid reaction mediators released by mast cells. Release curves were established for the anaphylactoid reaction mediators, and the release time, release breakthrough velocity, RI, and RRI of the anaphylactoid reaction mediators were defined to evaluate anaphylactoid reactions. The LAD2 release model was then employed to evaluate the anaphylactoid reactions induced by ciprofloxacin, norfloxacin, lomefloxacin, moxifloxacin, and baicalin. The results positively correlated with the Evans blue ear test and negatively correlated with Ca^2+^ influx EC_50_ values. In summary, the current study established a novel *in vitro* method to analyze the properties of histamine release from LAD2 cells and characterize the sensitization and strength of sensitization of drugs or components that induce anaphylactoid reactions. The RRI of histamine in LAD2 cells was used to evaluate the potential anaphylactoid effects of various drugs.

## Methods

### Reagents and chemicals

The LAD2 cell line was supplied by the American Type Culture Collection (ATCC, USA). The Ku812 cell line was supplied by the American Type Culture Collection (ATCC, USA). The HMC-1 cell line was supplied by the American Type Culture Collection (ATCC, USA). HPLC-grade methanol and acetonitrile were obtained from SK Chemicals Co., Inc. (Ulsan, Korea). All aqueous solutions were prepared using ultrapure water, which was produced using an MK-459 Millipore Milli-Q Plus ultra-pure water system. Compound 48/80, which is a condensation product of N-methyl-p-methoxyphenethylamine with formaldehyde, was used as a positive control to investigate histamine release.

### LC-MS/MS methods

Two Nexera LC-20AD_XR_ pumps, a SPD-20A UV/VIS detector, a CBM-20A communication bus module, an LCMS-8040 triple quadruple mass spectrometer, and a Lab Solutions work station (Shimadzu Corporation, Kyoto, Japan) were employed in the UPLC-ESI-MS/MS system. A UPLC column (Shim-pack XR-ODS II, 75 × 2.0 mm I.D., 3 μm, Shimadzu Corporation, Kyoto, Japan) was maintained at 37 °C in a CTO-20AC column oven. The following UPLC-ESI-MS/MS system conditions were used: a mobile phase of acetonitrile-water (0.5% acetic acid) (40:60, v/v) with a 0.4 mL min^−1^ flow rate and UV detection. The MS/MS conditions were: nebulizer gas (N_2_, purity > 99.999%), flow rate of 3.0 L min^−1^; drying gas (N_2_, purity > 99.999%), flow rate of 15.0 L min^−1^; interface, ESI source; desolvation line (DL) temperature, 250 °C; heat block temperature, 400 °C; interface voltage, 4.5 kV, interface current, 4.7 μA; detector voltage, 1.72 kV; CID gas (Ar, purity > 99.999%), pressure, 230 kPa; multiple reaction monitoring (MRM) mode. The detailed MRM method of histamine serotonin and PGE2 was shown in Supplementary Table 5. The detailed multiple reaction monitoring method of β-hexosaminidase and TNF-α after trypsin digestion was shown in Supplementary Table 6.

### Construction of release curves for five anaphylactic mediators

Time-effect release curves for five anaphylactic mediators were obtained using the LAD2, Ku812, and HMC-1 cell lines. Anaphylactic mediator release was induced by stimulating the cell lines with compound 48/80 as a model drug at different time points.

### *In vitro* histamine release curve

Histamine, the mediator released in the highest amounts, was confirmed to be the optimal mediator. Histamine release from LAD2 cells was selected to further evaluate anaphylactoid effects. Several new terms were defined as follows:

Release time (*t*): the time when the concentration of released histamine increased to 50% of the maximal concentration.

Release breakthrough velocity (*v*): the rate of histamine release speed after stimulation, calculated by equation (1):

1 $$V=\frac{{c}_{2}-{c}_{1}}{{t}_{2}-{t}_{1}}$$

where *c*_2_ is the final concentration of histamine after release, *c*_1_ is the initial concentration of histamine before release, *t*_2_ is the time the histamine concentration reached its maximum concentration, and *t*_1_ is the time that histamine release began.

*RI* is the acceleration speed of histamine release, calculated by equation (2):

2 $$RI=\frac{v}{t}$$

*RRI* is the RI of the ingredients compared with that of compound 48/80, calculated by equation (3):

3 $$RRI=\frac{R{I}_{otheringredients}}{R{I}_{compound48/80}}$$

### Mouse model

Mice were purchased from the Experimental Animal Center of Xi’an Jiaotong University (Xi’an, China). Adult male mice weighing 25–30 g were included in the study. All mice were housed in the Experimental Animal Center of Xi’an Jiaotong University. The mice were housed in individual cages in a large colony room with free access to water and were fed standard dry food twice per day. The breeding environment was maintained at 20–25 °C with a relative humidity of 40% and a day-night cycle of 12/12 h. All experiments involving equivalent treatments in animals were conducted by an experimenter blind to the conditions.

### Ethics statement

This study was carried out in strict accordance with the recommendations of the Guide for the Care and Use of Laboratory Animals from the National Institutes of Health. The experimental protocols for the mice were approved by the Animal Ethics Committee at Xi’an Jiaotong University, Xi’an, China (Permit Number: XJTU 2011-0045). All animals were operated on under chloral hydrate anesthesia.

### Hind paw swelling and histamine release assay

Experiments were carried out on adult male mice weighing 25–30 g that were anesthetized with an intraperitoneal injection of 0.08 mL of 10% chloral hydrate. Fifteen minutes after the induction of anesthesia, the thickness of the paw was measured with a Vernier caliper. After measurement, 5 µL of compound 48/80 (10 µg mL^−1^) was administered by a micro-injector in the left paw; saline was administered in the right paw as a negative control. Paw thickness was measured and recorded at specific time points (0, 5, 10, 15, 20, 25, and 30 min). For the histamine release assay, the mice were randomly assigned into 6 groups (5, 10, 15, 20, 25, and 30 min for the six groups) after anesthesia was administered. Fifteen minutes later, compound 48/80 (10 µg mL^−1^, 5 µL) was injected into the left paw and saline (5 µL) into the right paw; the mice were then sacrificed in sequence at specific time points, and all paw tissues were weighed and collected into 1.5-mL tubes. One milliliter of saline was added to each tube and allowed to stand on ice for 30 min; paw tissues were then cut into pieces, treated supersonically for 30 min, and centrifuged for 10 min at 10000 rpm and 4 °C. Fifty microliters of supernatant from each sample was collected into a clean 1.5-mL tube, mixed with 100 µL of *d*_4_-HA, and centrifuged for 10 min at 10000 rpm, 4 °C. Finally, 50 µL of supernatant was collected for histamine determination according to the previously described MRM method.

### Hind paw Evans blue extravasation studies

Evans blue extravasation studies were performed on adult male mice weighing 25–30 g that were anesthetized with an intraperitoneal injection of 0.08 mL of 10% chloral hydrate. Fifteen minutes later, each mouse was injected intravenously (i.v.) with 0.2 mL of 0.15% Evans blue in saline. The mice were randomly classified into 6 groups and placed back in separate cages (5, 10, 15, 20, 25, and 30 min for the six groups), after which the mice were administered 5 μL of compound 48/80 (10 µg mL^−1^) by a micro-injector in one paw and saline (5 µL) in the other paw. At specified time points (5, 10, 15, 20, 25, and 30 min), the mice were killed by decapitation, and a photo of each paw was taken; tissues were then collected into tubes, dried and weighed.

The dried paw tissues were soaked in 500 µL of acetone-saline (7:3, v/v) and cut into pieces; the total Evans blue content was obtained by sonicating for 30 min and incubating overnight at 37 °C. Each tissue solution was centrifuged at 10000 rpm for 10 min; 200 µL of the supernatant was transferred into a 96-well plate in sequence, and the OD values were read at 620 nm using a spectrophotometer.

### Histamine release during systemic anaphylaxis reactions

Adult male mice weighing 25–30 g were employed to carry out a systemic histamine release assay. Animals were randomly assigned to 6 groups and maintained in separate cages. Mice were given an i.v. injection of compound 48/80 (400 µL per 20 g of weight) in saline (0.125 mg mL^−1^), and saline was used as the negative control. At specific time points (5, 10, 15, 20, 25, and 30 min), blood was collected after removing the eye, and the blood samples were left to stand at 4 °C for 1 h; then, 50 µL of supernatant (serum) was obtained after centrifugation at 12000 rpm for 20 min. Each serum sample collected was mixed with 100 µL of *d*_4_-HA and centrifuged for 10 min at 10000 rpm, 4 °C; 50 µL of supernatant was collected for HA analysis according the previously described MRM method.

### Intracellular Ca^2+^ mobilization assay

*Mrgpr*X2-HEK293 cells or NC-HEK293 cells were plated at 1 × 10^4^ cells per well in 96-well plates and incubated overnight at 37 °C with 5% CO_2_. All drug substances were diluted to the required concentration in calcium imaging buffer (CIB). The incubation buffer consisted of 0.8 µL of Fluo-3, 3 µL of pluronic F-127 and 996.2 µL of CIB. Cells were washed twice in 100 µL of CIB, and then 100 µL of incubation buffer was added for 40 min. The cells were washed twice and then used immediately for imaging. For calcium imaging, the cells were magnified 200 times, and one photo per second was taken under blue light. The cells were identified as responsive if the [Ca^2+^]^i^ rose by at least 50% after injection.

### siRNA transfection of LAD2 cells

Specific knockdown was achieved using small interfering siRNAs targeting *Mrgpr*X2 or a control siRNA. A smart pool of double-stranded siRNAs targeting *Mrgpr*X2, as well as non-specific siRNAs, was obtained from Shanghai Gene Pharma Co., Ltd. The siRNA sequences were as follows: forward, 5′-GUACAACAGUGAAUGGAAATT-′, and reverse, 5′-UUCCAUUCACUGUUGUACTT-′ for *Mrgpr*X2; and forward, 5′-UCUCCGAACGUGUCACGUTT-′, and reverse, 5′-CGUGACACGUUCGGAGAATT-′ for the control. For transfection, siRNA was delivered at a final concentration of 1 μmol L^−1^ using Lipofectamine® 2000 reagent according to the manufacturer’s instructions. The 1.5 × 10^5^ cells were incubated for 48 h to allow knockdown of *Mrgpr*X2. These cells were then used for calcium imaging.

### Evans blue ear extravasation studies

Ciprofloxacin hydrochloride, moxifloxacin, norfloxacin, and lomefloxacin were prepared in Evans blue saline solution at concentrations of 0.65, 1.30, and 2.60 mg mL^−1^; compound 48/80 was prepared at concentrations of 0.0625, 0.1250, and 0.25 mg mL^−1^; and baicalin was dissolved in Evans blue sodium bicarbonate solution at concentrations of 0.65, 1.30, and 2.60 mg mL^−1^.

Adult male mice weighing 25–30 g were used to perform the extravasation studies. Animals were randomly assigned to the control group (saline group and sodium bicarbonate group) and drug groups. All tested drugs at different concentrations (200 µL) and the negative control solutions (200 µL) were administered intravenously. One hour later, the mice were sacrificed by decapitation, and a photo of each ear was taken. Both ears from each mouse were placed in one tube, and the tissues of all mice were collected for Evans blue extraction using 300 µL of acetone-saline (7:3, v/v), consistent with hind paw Evans blue extravasation studies.

## Electronic supplementary material

Supplementary tables and figures
