# Supplementary material for: Use of the relative release index for histamine in LAD2 cells to evaluate the potential anaphylactoid effects of drugs
Source: Sci Rep. 2017 Oct 20;7:13714. doi: 10.1038/s41598-017-14224-z (PMC5651870; doi:10.1038/s41598-017-14224-z)

Use of the relative release index for histamine in LAD2 cells to evaluate the potential anaphylactoid effects of drugs

Shengli Han[1, 3†], Yanni Lv[1†], Liyun Kong[1], Delu Che[1], Rui Liu[1], Jia Fu[1], Jiao Cao[1], Jue Wang[1], Cheng Wang[1], Huaizhen He[1], Tao Zhang[1], Xinzhong Dong[2*], and Langchong He[1]*

[1] *School of Pharmacy, Xi'an Jiaotong University, Xi'an 710061, China.* [2] *The Solomon H. Snyder Department of Neuroscience, Johns Hopkins University, School of Medicine, Baltimore, Maryland 21205, USA.* [3] *Department of Pharmaceutical Science, School of Pharmacy, University of Wisconsin-Madison, Madison, WI 53705, USA.*

† These two authors contributed equally to this work.

*Corresponding authors: Langchong He and Xinzhong Dong

Addresses for correspondence:

School of Pharmacy, Xi’an Jiaotong University, Xi’an 710061, China.

The Solomon H. Snyder Department of Neuroscience, Johns Hopkins University, School of Medicine, Baltimore, Maryland 21205, USA.

E-mail: helc@mail.xjtu.edu.cn; xdong2@jhmi.edu

Tel.: +86-29-82656788;

Fax: +86-29-82655451;

Running title: LAD2 cell release model.

Supplementary Table.1 The system suitability of LC-MS/MS method used to analyze the anaphylactoid reaction mediators released by mast cell.

Validation criterion	Precision						LOD (ng/mL)	LOQ (ng/mL)	Linearity range (ng/mL)	Accuracy (% Recovery)
	Intra-day (n=5)			Inter-day (n=5)						
	2ng/mL	10ng/mL	50ng/mL	2ng/mL	10ng/mL	50ng/mL				
Serotonin	7.69%	3.70%	3.76%	6.35%	5.26%	2.46%	1.0	3.0	3-60	92.15-106.44%
Thromboxane	6.69%	4.22%	2.96%	6.15%	4.39%	3.08%	2.0	6.0	6-50	88.73-110.24%
PGE2	7.18%	5.31%	3.06%	5.14%	3.97%	2.88%	1.3	4.0	4-50	90.63-104.27%
PGD2	5.36%	4.29%	2.87%	5.28%	4.13%	3.69%	1.3	4.0	4-50	92.11-105.32%
Methyl Histamine	8.40%	6.36%	2.39%	7.76%	3.43%	7.03%	0.67	2.0	2-50	83.60-112.42%
Histamine	4.56%	4.56%	3.11%	7.94%	4.70%	3.63%	0.67	2.0	2-50	93.38-108.25%
Leukotriene E4	6.21%	4.22%	3.84%	5.36%	3.52%	2.54%	2.0	6.0	6-50	88.69-107.32%
PAF	5.84%	4.64%	3.91%	6.11%	4.12%	3.16%	2.0	6.0	6-50	90.22-110.68%
IL-4	7.95%	5.66%	4.22%	6.82%	5.34%	2.98%	2.0	6.0	15-100	92.58-108.41%
IL-6	9.16%	6.71%	4.38%	7.35%	4.26%	3.17%	5.0	15.0	15-100	93.46-107.18%
IL-8	8.92%	6.13%	4.71%	6.55%	3.64%	3.41%	5.0	15.0	15-100	86.49-108.29%
β-hexosaminidase	8.42%	6.22%	5.16%	7.51%	4.72%	3.62%	6.7	20.0	20-100	90.63-107.56%
TNF-α	7.93%	5.88%	4.91%	8.06%	3.97%	4.13%	6.7	20.0	20-100	85.46-109.23%

Supplementary Table.2 the release time, increase rate, and release index of LAD2, Ku812, and HMC-1 cells stimulated by compound 48/80.

		LAD2	Ku812	HMC-1
t (min)	Histamine	15.1	16.2	18.3
	Serotonin	19.6	18.2	23.8
	PGE2	26.6	31.2	32.5
	β-hexosaminidase	27.6	32.8	36.6
	TNF-α	30.6	35.1	40.2
v (mol/min)	Histamine	9.5×10^{-11}	5.2×10^{-11}	1.9×10^{-11}
	Serotonin	5.9×10^{-12}	5.1×10^{-12}	2.4×10^{-12}
	PGE2	2.1×10^{-12}	1.9×10^{-12}	0.8×10^{-12}
	β-hexosaminidase	2.2×10^{-14}	1.6×10^{-14}	0.9×10^{-14}
	TNF-α	6.5×10^{-15}	3.8×10^{-15}	0.8×10^{-15}
Release Index（mol/min^2）	Histamine	6.29×10^{-12}	3.21×10^{-12}	1.04×10^{-12}
	Serotonin	3.01×10^{-13}	2.80×10^{-13}	1.01×10^{-13}
	PGE2	7.89×10^{-14}	6.09×10^{-14}	2.46×10^{-14}
	β-hexosaminidase	7.97×10^{-16}	4.88×10^{-16}	2.46×10^{-16}
	TNF-α	2.12×10^{-16}	1.08×10^{-16}	0.20×10^{-16}

Supplementary Table.3 The subtypes of LAD2, Ku812, and HMC-1 cells.

	LAD2	Ku812	HMC-1
IgE-R	α β γ	α β γ	β γ
Mrgpr	X2	-	-

Supplementary Table.4 Histamine release characteristics and EC_{50} values of the sixpotential allergic components with compound 48/80 as positive control.

		Compound48/80	Ciprofloxacin	Norfloxacin	Lomefloxacin	Moxifloxacin	Baicalin
t(min)		14.8	15.6	16.4	17.3	19.2	22.6
v(mol/min)		1.07×10^{-10}	9.70×10^{-11}	8.91×10^{-11}	6.85×10^{-11}	4.24×10^{-11}	2.07×10^{-11}
Release Index （mol/min^2）		7.15×10^{-12}	6.22×10^{-12}	5.43×10^{-12}	3.96×10^{-12}	2.21×10^{-12}	0.92×10^{-12}
Relative Release Index		1	0.93	0.81	0.66	0.31	0.13
EC50（μM）		2.5±1.2	6.4±1.12	24.3±3.88	46.2±9.36	86.8±13.36	165.1±120.24
	Low	0.18±0.015	0.15±0.014	0.12±0.012	0.09±0.031	0.05±0.008	0.01±0.016
OD Values	Middle	0.26±0.017	0.24±0.016	0.19±0.007	0.15±0.022	0.09±0.014	0.05±0.012
	High	0.31±0.022	0.28±0.017	0.24±0.030	0.21±0.031	0.14±0.010	0.10±0.015

Supplementary Table.5 The detailed multiple reaction monitoring method of histamine serotonin and PGE2.

mediators	molecular weight	ion pair (*m/z*)	ionization mode	collision Energy
histamine	111.15	111.90 > 95.15	+	-15.0
serotonin	176.18	177.00 > 160.10	+	-11.0
PGE_2	352.47	351.00 > 271.20	-	17.0

Supplementary Table.6 The detailed multiple reaction monitoring method of β-hexosaminidase and TNF-α after trypsin digestion.

mediators	unique peptide	ion pair (*m/z*)	ionization mode	collision energy
β-hexosaminidase	LAPGTIVEVWK	606.85 > 514.80*	+	-19.0
	IQDFMR	441.25 > 651.35*	+	-15.7
	MVIEYAR	405.20 > 568.25*	+	-15.9
TNF-α	ANALLANGVELR	620.85 > 758.42*	+	-20.3
	VNLLSAIK	429.28 > 758.48*	+	-17.7
	IAVSYQTK	455.26 > 725.38*	+	-16.7

Supplementary Figure 1. The effect of MrgprX2 knockdown by siRNA in LAD2 cells. (A) The MrgprX2 mRNA expression in LAD2 cells transfected with 1 μM Negative Control (NC) or MrgprX2 siRNA respectively using Lipofectamine® 2000. (B) Protein expression levels of MrgprX2 in the LAD2 cells transfected with siRNA. (C) Quantification of MrgprX2 protein expression by densitometric analysis. The data are presented as mean ± S.D. (n=3). Two-tailed unpaired Student's t-test was used to determine significance in statistical comparisons, and statistical significance was accepted at $p<0.05$ (*$p<0.05$, **$p<0.01$).

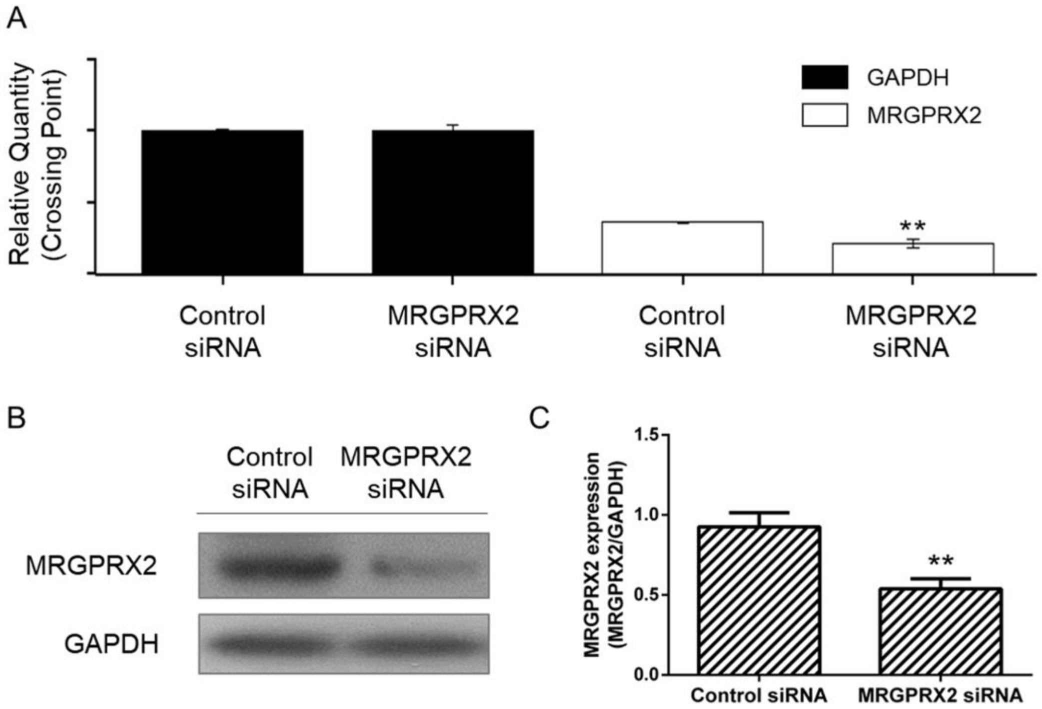

Supplementary Figure 2. Calcium image analysis interactions relationships between substances and MrgprX2. The Ca^{2+} influx in HEK293 cells induced by substances. (Compound 48/80, ciprofloxacin, norfloxacin, moxifloxacin, lomefloxacin, griseofulvin, methacycline, sodium penicillin G, cefradine, emodin, baicalin)

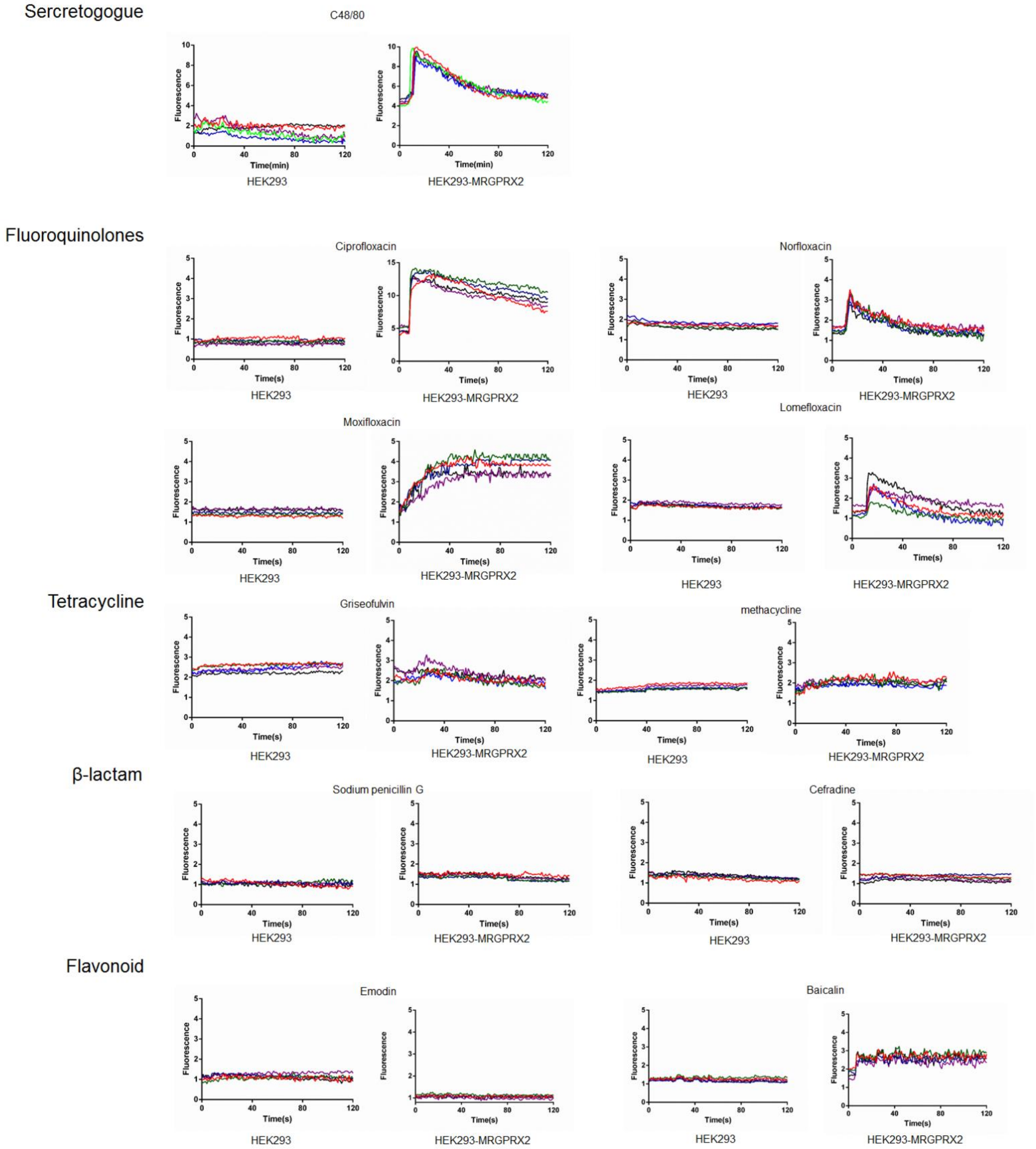

Supplement: Supplementary file 1 — Supplementary tables and figures [file 41598_2017_14224_MOESM1_ESM.pdf]
